# Supplementary material for: COVID-19 Vaccination in Developing Nations: Challenges and Opportunities for Innovation
Source: Infect Dis Rep. 2021 May 14;13(2):429–36. doi: 10.3390/idr13020041 (PMC8162348; doi:10.3390/idr13020041)
Supplement: Supplementary file 1 [file idr-13-00041-s001.zip › idr-1200762-supplementary.pdf]

Supplementary Table S1:

Vaccine name/ Manufacturer	Age (in years)	Recommended duration be-tween 1st & 2nd dose	Vaccine efficacy*	Use in COVAX	Storage Recommendations	FDA approved	WHO Emergency use listing	Affordability
Bharat Biotech [1]	≥18 [2]	28 days [3]	81.0% [3]	No [1]	2-8°C [1]	No [4]	No	$ [1]
Novavax [1]	≥12 [5]	21 days [5]	96.4% [6]	Yes [1]	2-8°C [1]	No [4]	No	$ [1]
CanSino [1]	≥18 [6]	Single dose [6]	90.9%; 65.7% for 1 dose [7]	No [1]	2-8°C [1]	No [4]	No	$$ [8]
QazCovid-in [9]	≥18 [9]	21 days [9]	96.0% [10]	No [1]	2-8°C [10]	No [4]	No	$ [11]

***vaccine efficacy**: as noted in phase 3 trials, real world data may vary, **COVAX**-COVID-19 Vaccines Global Access-an international alliance led by Coalition for Epidemic Preparedness Innovations (**CEPI**), Gavi, the Vaccine Alliance (**GAVI**), World Health Organization (**WHO**) and United Nations Children's Fund (**UNICEF**), **FDA**- U.S. Food and Drug Administration, **Affordability:** Based on lowest offered price in US dollars; <USD 10 - $, USD 10-30 - $$, >USD 30 - $$$

References:

1. Wouters, O.J.; Shadlen, K.C.; Salcher-Konrad, M.; Pollard, A.J.; Larson, H.J.; Teerawattananon, Y.; Jit, M. Challenges in ensuring global access to COVID-19 vaccines: production, affordability, allocation, and deployment. *Lancet.* **2021**, *397*, 1023-1034, doi: 10.1016/S0140-6736(21)00306-8.

2. An Efficacy and Safety Clinical Trial of an Investigational COVID-19 Vaccine (BBV152) in Adult Volunteers. ClinicalTrials.gov. 2021. Available from: https://clinicaltrials.gov/ct2/show/NCT04641481. (Accessed on 5 May 2021)

3. Sputnik V, Covishield, Covaxin: What we know about India's Covid-19 vaccines. BBC News. 2021. Available from: https://www.bbc.com/news/world-asia-india-55748124#:~:text=Bharat%20Biotech%20is%20a%20Hyderabad-based%20pharmaceutical%20company.%20The,preliminary%20data%20from%20its%20phase%203%20trial%20shows. (Accessed on 5 May 2021)

4. COVID-19 vaccines. US Food and Drug Administration. 2020. Available from: https://www.fda.gov/emergencypreparedness-and-response/coronavirus-disease-2019-covid-19/covid-19-vaccines. (Accessed on 3 April 2021)

5. A Study to Evaluate the Efficacy, Immune Response, and Safety of a COVID-19 Vaccine in Adults ≥ 18 Years With a Pediatric Expansion in Adolescents (12-17 Years) at Risk for SARS-CoV-2. ClinicalTrials.gov. 2021. Available from: https://clinicaltrials.gov/ct2/show/NCT04611802. (Accessed on 5 May 2021)

6. Phase III Trial of A COVID-19 Vaccine of Adenovirus Vector in Adults 18 Years Old and Above. ClinicalTrials.gov. Available from: https://clinicaltrials.gov/ct2/show/NCT04526990. (Accessed on 5 May 2021)

7. China's CanSino Covid Vaccine Shows 65.7% Efficacy. Bloomberg News. 2021. Available from: https://www.bloomberg.com/news/articles/2021-02-08/pakistan-says-cansino-s-covid-vaccine-shows-65-7-efficacy. (Accessed on 5 May 2021)

8. Govt prefers free-of-cost Covid vaccine, PAC told. The Express Tribune. 2021. Available from: https://tribune.com.pk/story/2287563/govt-prefers-free-of-cost-covid-vaccine-pac-told. (Accessed on 5 May 2021)

9. Immunogenicity, Efficacy and Safety of QazCovid-in® COVID-19 Vaccine. ClinicalTrials.gov. 2021. Available from: https://clinicaltrials.gov/ct2/show/NCT04691908. (Accessed on 5 May 2021)

10. Where and how Kazakhstan's homegrown QazCovid-in vaccine is developed. Kazinform. 2021. Available from: https://www.inform.kz/en/where-and-how-kazakhstan-s-homegrown-qazcovid-in-vaccine-is-developed_a3776423. (Accessed on 5 May 2021)

11. Kazakhstan vaccine against CVI to be rolled out in late April. Available from: https://www.kazpravda.kz/en/news/society/kazakhstan-vaccine-against-cvi-to-be-rolled-out-in-late-april. (Accessed on 5 May 2021)
